# Dual Antioxidant Activity: Preventive and Scavenger Effects of Wild and Cultivated *P. nebrodensis* Extracts Against ROS and SOX in Human Keratinocytes

**DOI:** 10.3390/antiox14121439

**Published:** 2025-11-28

**Authors:** Daniela Ratto, Maria Teresa Venuti, Anthea Desiderio, Ilenia Cicero, Gaetano Balenzano, Elena Savino, Giuseppe Venturella, Maria Letizia Gargano, Paola Rossi

**Affiliations:** 1 Department of Biology and Biotechnology “L. Spallanzani”, University of Pavia, 27100 Pavia, Italy; daniela.ratto@unipv.it (D.R.); mariateresa.venuti01@universitadipavia.it (M.T.V.); 2 Department of Earth and Environmental Sciences, University of Pavia, 27100 Pavia, Italy; anthea.desiderio01@universitadipavia.it (A.D.); elena.savino@unipv.it (E.S.); 3 Department of Agricultural, Food and Forest Sciences, University of Palermo, 90128 Palermo, Italy; ilenia.cicero01@unipa.it (I.C.); giuseppe.venturella@unipa.it (G.V.); 4 Department of Soil, Plant and Food Sciences, University of Bari “Aldo Moro”, Campus Universitario “E. Quagliariello”, 70125 Bari, Italy; gaetano.balenzano@uniba.it

**Keywords:** *P. nebrodensis*, antioxidants, ROS, SOX, scavenger activity, human keratinocytes, skin, α-glucans, β-glucans

## Abstract

*Pleurotus nebrodensis*, a rare endemic Sicilian mushroom with notable gastronomic and medicinal value, attracts interest for its potential antioxidant properties, though data on its biological effects in skin models are lacking. This study evaluated the antioxidant activities of several aqueous extracts from wild (1a, 2a, 3a, 1b, 2b, and 3b) and cultivated (CAN1 °F, 3A, 2B(II), and CAN2 °F) *P. nebrodensis* basidiomes in human keratinocytes (HaCaT cells). Extracts were characterized through DPPH radical scavenging assay, MTT viability assay, and intracellular ROS and mitochondrial SOX quantification by DCFH-DA and MitoSOX Red fluorescence analyses. The methodology specifically included two approaches in keratinocytes: co-treatment of extracts and H_2_O_2_ to investigate direct scavenger activity, and pre-treatment to assess the preventive activity on oxidative stress modulation. This analysis demonstrated that selected extracts (1b and CAN2 °F) exert a dual action, combining anti-intracellular ROS and anti-mitochondrial SOX preventive effect with a direct free radical scavenging activity in human keratinocytes. In particular, CAN2 °F exerts its activity predominantly through prevention (modulation of cellular defenses), while 1b primarily functions as a direct intracellular ROS and mitochondrial SOX scavenger. Notably, glucan quantification revealed a correlation between β-glucan content and the overall antioxidant activity. These findings provide the first evidence of *P. nebrodensis*’s anti-ROS and anti-SOX efficacy in human keratinocytes, highlighting its potential as a source of natural bioactives for cosmeceutical and dermatological applications.

## 1. Introduction

Oxidative stress, caused by an imbalance between reactive oxygen species (ROS) production and antioxidant defenses, plays a critical role in cellular damage and skin aging. Human keratinocytes (e.g., the HaCaT cell line) are highly sensitive to oxidative insults, which can impair epidermal barrier function and promote premature aging or pathological alterations [1]. Beyond aging, oxidative stress is also implicated in chronic skin conditions such as psoriasis, atopic dermatitis, and carcinogenesis, further emphasizing the biomedical importance of antioxidant protection in keratinocytes [2,3,4]. Therefore, strategies to protect keratinocytes from oxidative stress are crucial from both cosmetic and biomedical perspectives.

In this context, medicinal mushrooms are well-recognized as rich sources of bioactive compounds with antioxidant properties [5]. While species such as *Ganoderma lucidum* (Curtis) P. Karst. and *Lentinula edodes* (Berk.) Pegler have been widely studied for their antioxidant capacity, rare taxa remain largely unexplored [6,7]. Among them, *Pleurotus nebrodensis* (Inzenga) Quèl. (Pleurotacea) is a rare, endemic, declining species from Sicily, historically celebrated as “the most delicious mushroom of the Sicilian mycological flora” [8]. It belongs to the so-named “*Pleurotus eryngii* species complex” and to the ecological category of saprotrophs, growing on the dead roots of *Prangos ferulacea* (L.) Lindl. (Apiaceae) between April and June on the Madonie Mts. (Sicily) [9]. The *P*. *nebrodensis* basidiomes exhibit good nutritional value and interesting vitamin contents [10,11]. Commercial strains from Asian countries labeled as “*P*. *nebrodensis*” have been characterized as a different taxon, *P*. *eryngii* subsp. *tuoliensis* [12], demonstrating that the “true” *P. nebrodensis* grows exclusively in the Mediterranean area. Due to overharvesting, including the collection of unripe basidiomes, and habitat degradation, *P. nebrodensis* has been classified as Critically Endangered (CR) on the IUCN Red List and, following a finding in Greece, is now listed as an Endangered (EN) species [9].

Investigating its bioactive potential is therefore not only of pharmacological interest but may also support biodiversity conservation efforts, highlighting the loss of therapeutic opportunities linked to species decline.

Beyond its prized gastronomic reputation, recent in vitro studies have revealed that extracts of *P. nebrodensis* exhibit promising medicinal properties. Specifically, these extracts have been shown to inhibit the proliferation of colon, sarcoma, and breast cancer cells through the induction of apoptosis [13,14,15], and to exert antibacterial activity against medically relevant bacterial strains [16]. These findings position *P. nebrodensis* as a high-value culinary-medicinal mushroom with significant pharmacological interest.

Nevertheless, despite this promising evidence, its antioxidant potential remains poorly characterized, especially under conditions of skin oxidative stress. To our knowledge, no previous studies have assessed the antioxidant effects of *P. nebrodensis* extracts in human keratinocytes, a relevant model for studying skin physiology and oxidative stress. Given the growing scientific and industrial interest in mushroom-derived antioxidants for dermatological applications, filling this gap is timely and necessary. Accordingly, this study pioneers the evaluation of the reactive oxygen species (ROS)- and superoxide (SOX)-related antioxidant activity of different *P. nebrodensis* strains in vitro in keratinocyte cells. This research article provides the first evidence of the antioxidant activity of *P. nebrodensis* through two different cellular mechanisms, e.g., preventive and scavenger activities, in human keratinocytes. These findings not only establish the novelty of our work but also open new perspectives for the development of *P. nebrodensis*-based cosmeceutical, nutraceutical, and pharmacological applications.

## 2. Materials and Methods

### 2.1. Sample Collection and Identification

In the years 2024–2025, field excursions were conducted in the Madonie Mountains (North Sicily, Italy) on pastures dominated by *Prangos ferulacea* (L.) Lindl., during the fruiting period of *P. nebrodensis* (late April to mid-June). Six basidiomata labeled as 1a, 2a, 3a, 1b, 2b, and 3b were collected in the locality of Piano Battaglia (Madonie Mts.), 37°52′39″ N, 14°02′06 E″, 1687 m a.s.l. These six samples will be referred to as wild-type (WT).

The specimens were identified, dried, and preserved at the Department of Soil, Plant and Food Sciences (Di.S.S.P.A.), University of Bari Aldo Moro, Bari (Italy). The macro- and micromorphological identification was carried out using a Leica MS5 binocular microscope, as described by Venturella et al. (2016) [12]. Macroscopic features include: size, shape, and color of the cap; shape and surface of the margin; surface and flesh of the cap; lamellar characteristics; stem characteristics; presence or absence of a veil on the stem; type of basidioma attachment; and spore print. Colors refer to the RAL matching system (RAL is a color matching system that defines colors for paints, coatings, and plastics; the RAL standard is managed by the RAL Deutsches Institut für Gütesicherung und Kennzeichnung, based in Bonn, Germany). The microscopic characteristics evaluated were: hyphal system; hyphal wall; septa; hyphal branching; hyphal inflations; specialized hyphae; pigmentation; type of pellis; texture, position, type, shape, pigmentation, and encrustations of cystidia; basidia and basidioles; spore characteristics; chemical reactions. The nomenclature of vascular plants follows POWO (Plants of the World Online), while the nomenclature of fungi is referred to Index Fungorum.

### 2.2. Establishment of Pure Cultures and Preparation of Cultivation Bags

The mycelium of the wild-type *P. nebrodensis* basidiomes was isolated in pure culture as follows: a piece of pseudo-tissue was removed from the fresh basidoma, placed on potato dextrose agar (PDA) in Petri dishes under aseptic conditions, and incubated for 15 days at 25 ± 2 °C. Sterile portions of wild-type mycelia (1a, 2a, 3a, 1b, 2b, and 3b) were inoculated to obtain actively growing mycelium, known as spawn. The mycelium growth takes ca. 45 days.

The pure culture was provided to Italmiko (Senise, Potenza, Italy) for inoculation into mushroom substrates composed of moistened wheat straw and sugar beet. Then, the substrate was transferred to a mixing and dosing EVERSUN mushroom substrate belt mixer and packaged in heat-resistant bags weighing 4 kg. Sterilization was performed in an autoclave, twice at 120 °C, 1.1 Atm for 20 min with a 24 h interval between each cycle. After the final cooling, substrates were inoculated using the spawn and incubated at 25 °C in the dark. The incubation took ca. 90 days.

Six wild-type strains were employed for indoor cultivation, resulting in four cultivated strains, as reported in Table 1.

The indoor cultivation of the bags inoculated with the strains of *P*. *nebrodensis* (strains 3A, 2B(II), CAN1 °F, CAN2 °F), was carried out in a farm located in Gangi (Madonie Mts., northern Sicily) at an altitude of ca. 1000 m.

### 2.3. Preparation of P. nebrodensis Mushroom Powder

A total of 1 kg of basidiomes collected in the cultivation tunnels was thinly sliced and dried in a patented mushroom-drying appliance (Valla dryer, Borgotaro, Emilia-Romagna, northern Italy) equipped with baskets for 24 h. Then, the dried mushroom slices were placed inside a Bimby TM7 Multifunction Kitchen Robot to obtain a fine powder for future biological and chemical analysis.

### 2.4. Extraction Procedure

For the aqueous extraction protocol, we adopted the method described by Tripodi et al. (2022) [17] as a starting point. Subsequently, the protocol was modified to optimize and maximize the extraction yield for the fungal species tested. Samples were subjected to 3–4 freeze-thaw cycles at −80 °C to promote cell disruption. Then, 1 g of dried basidiomes was suspended in 10 mL of Milli-Q water in an open flask and extracted under continuous stirring (500 rpm) at 50 °C for 3 h. The resulting mixture was centrifuged at 5000 rpm for 30 min, and the supernatant was collected, stored at −20 °C overnight, and lyophilized for 24 h. After 24 h, the freeze-dried sample was weighed, and the extraction yield was calculated (Table 2 and Table 3).

### 2.5. DPPH (2,2-Diphenyl-1-Picryl-Hydrazil) Radical Scavenging Assay

The antioxidant activity of the *P. nebrodensis* extracts was evaluated using the 2,2-diphenyl-1-picrylhydrazyl (DPPH) radical scavenging assay, following the procedure described by Desiderio et al. (2025) [18]. Briefly, 30 µL of eight serial dilutions (from 0.016 to 2 mg/mL) prepared from the extract stock solution (20 mg/mL) were mixed with 270 µL of a methanolic DPPH solution (6 × 10^−5^ mol/L) in each well. The reaction mixtures were incubated in the dark for 60 min at room temperature, after which the absorbance was recorded at 515 nm, using a SpectraMax ID5 spectrophotometer (Molecular Devices, San Jose, CA, USA).

Results were expressed as IC_50_ values (mg/mL), representing the extract concentration required to reduce the initial DPPH concentration by 50%. Ascorbic acid (Farmacia Fapa, Pavia, Italy) was used as a positive control at concentrations ranging from 500 μM to 1 μM, and the experiments were performed in triplicate.

### 2.6. HaCaT Cell Line

The immortalized human keratinocyte HaCaT cell line was obtained from Cell Line Service (Eppelheim, Germany; catalog number: 300493). HaCaT cells were cultured in Dulbecco’s Modified Eagle Medium (DMEM) supplemented with 10% fetal bovine serum (FBS) and 1% penicillin-streptomycin, and maintained at 37 °C in a humidified incubator with 5% CO_2_. Upon reaching about 90% confluence, cells were harvested using TrypLE Express and replated in new flasks with fresh growth medium. All the materials were purchased from CLS and Gibco—Life Technologies Europe BV, Bleiswijk, the Netherlands. For the MTT [3-(4,5-dimethylthiazol-2-yl)-2,5-diphenyltetrazolium bromide] assay, HaCaT cells were seeded into a 96-well plate at a density of 10.000 cells per well (200 µL), for DCFH-DA (2′,7′-Dichlorodihydrofluorescein Diacetate) and MitoSOX Red assay, 150,00 HaCaT cells were seeded on coverslips (22 × 22 mm). Then, cells were incubated overnight at 37 °C with 5% CO_2_ to allow cell adhesion.

### 2.7. Cell Viability Evaluation: MTT Assay

To evaluate the effect of 24 h of treatment with different *P. nebrodensis* extracts on HaCaT cells viability, the MTT assay was performed in accordance with Brandalise et al. (2025) [19].

Briefly, after seeding, the culture medium was replaced with a fresh medium supplemented with different extract concentrations (0 for the control condition and ranging from 0.03 to 2 mg/mL). After 24 h of treatment, MTT solution (0.5 mg/mL) was added for 3 h at 37 °C. Next, dimethyl sulfoxide (DMSO) was used to dissolve the formazan crystals, and the absorbance of the samples at 550 nm was evaluated using the ELx808TM Absorbance Microplate Reader (Bio-Tek Instruments, Inc., Winooski, VT, USA). From the absorbance data, we obtained the percentage of cell viability that allows us to calculate the IC_25_ value, meaning the concentration necessary to inhibit 25% of cell viability. In particular, the IC_25_ value was derived from the MTT viability curve for each extract, ensuring concentration was chosen based on its unique cell viability profile. All the experiments were conducted at least in triplicate, and the vehicle (H_2_O) did not show any difference compared to the control.

### 2.8. Evaluation of Antioxidant Activity: DCFH-DH Assay

To evaluate the antioxidant action of *P. nebrodensis* extracts in HaCaT cells, the DCFH-DA (2′,7′-Dichlorodihydrofluorescein Diacetate) assay was performed in accordance with Desiderio et al. (2025) [18]. After seeding, two experimental approaches were applied to assess the effects of the extracts: (i) pretreatment (24 h exposure to the extracts—preventive antioxidant effect), and (ii) co-treatment (30 min exposure—direct scavenging activity).

For the pretreatment condition, after the cell adhesion, cells were incubated for 24 h with the IC_25_ concentration of selected extracts before oxidative stress induction. Subsequently, two experimental conditions were established: (i) basal and (ii) oxidative stress induction. For the basal state, after the treatment, cells were directly incubated with 10 µM DCFH-DA for 30 min. In contrast, for the oxidative stress state, after the treatment, cells were washed with phosphate-buffered saline (PBS) 1× and then exposed to 500 µM hydrogen peroxide (H_2_O_2_) for 30 min, followed by incubation with 10 µM DCFH-DA for 30 min. In particular, this regimen (500 µM H_2_O_2_ for 30 min) was chosen in preliminary experiments to avoid any cytotoxicity in the HaCaT cell line. For the direct scavenging condition, both basal and oxidative stress states were reproduced as above, with the difference that cells were treated simultaneously with the extracts (IC_25_) and H_2_O_2_ (500 µM) for 30 min, and then incubated with 10 µM DCFH-DA for 30 min.

After incubation with DCFH-DA, in all conditions, cells were washed three times with PBS 1× to remove residual probe, and fluorescence intensity (excitation/emission wavelengths of 490/516) was immediately measured. Fluorescence, directly reflecting intracellular ROS production, was detected using a Leica DM6B WF microscope (Leica Microsystems, Buccinasco, MI, Italy). Images were acquired with an ORCA-Flash4.0 V3 Digital CMOS camera C13440-20CU (Hamamatsu Photonics, Arese, MI, Italy) and processed with Leica Application Suite X (LAS X, Version 5.1.0). ROS levels were quantified using ImageJ software (version 1.46p, NIH, Bethesda, MA, USA). The ROS levels are presented as the percentage relative to the basal untreated HaCaT cells (basal control), which were set as 100%. All the experiments were conducted at least in triplicate.

### 2.9. Reconstruction of Total Ranking

The results obtained from the chemical (DPPH) and cytological (DCFH-DA) assays were normalized by setting the highest value observed in each assay to 100, with all other values rescaled proportionally. Therefore, each sample received a normalized score (0–100) for both assays. The normalized scores were then summed to obtain a cumulative antioxidant activity index. This index was used to establish the total ranking, with higher values indicating stronger overall activity.

### 2.10. Glucan Quantification: Megazyme Yeast and Mushroom Kit

Mushroom extracts were analyzed for 1,3:1,6 β-glucan content using the Megazyme yeast and mushroom kit (K-YBGL) (Megazyme Ltd., Bray, Co. Wicklow, Ireland). Assays were carried out according to the manufacturer’s instructions. Briefly, total glucan content was quantified through controlled acid hydrolysis using sulfuric acid (H_2_SO_4_), followed by enzymatic treatment with a mixture of exo-1,3-β-glucanase and β-glucosidase. The amount of glucose released was subsequently determined using a glucose oxidase-peroxidase (GOPOD) reagent. The α-glucan fraction was specifically assessed by enzymatic hydrolysis of starch-like glucans with glucoamylase, after which the liberated glucose was quantified using the same GOPOD method. The β-glucan content was calculated as the difference between total glucan-derived glucose obtained after acid hydrolysis and the α-glucan-derived glucose from specific enzymatic hydrolysis. For both total and α-glucan determinations, measurements of D-glucose originating from oligosaccharides, sucrose, and free glucose were included. Final glucan values were expressed as a percentage of the sample dry weight [20]. The experiments were conducted in quadruplicate.

### 2.11. Evaluation of Antioxidant Activity: MitoSOX Red Assay

The fluorogenic dye MitoSOX was used to assess the mitochondrial superoxide in HaCaT cells. HaCaT cells were used in the DCFH-DA assay, and the same conditions and states were used. Two experimental approaches were applied to assess the effects of the extracts: (i) 24 h of pretreatment and (ii) 30 min of co-treatment, and for each approach, two experimental conditions were established: (i) basal and (ii) oxidative stress induction.

For the detection of superoxide (SOX), cells were incubated with MitoSOX Red 5 µM and MitoTracker Green 200 nM in HBSS with Ca^2+^ and Mg^2+^, for 30 min at 37 °C in the dark. Subsequently, for all conditions, cells were washed three times with HBSS containing Ca^2+^ and Mg^2+^, and the fluorescence was acquired within 1 h (MitoSOX: excitation/emission wavelengths of 510/580; MitoTracker Green: excitation/emission wavelengths of 490/516). In particular, the fluorescence was examined using a Leica DM6B WF microscope (Leica Microsystems, Buccinasco, MI, Italy). The images were captured using an ORCA-Flash4.0 V3 Digital CMOS camera (C13440-20CU; Hamamatsu Photonics, Arese, MI, Italy) and recorded with the Leica Application Suite X (LAS X; Version 5.1.0). Next, SOX levels were measured using ImageJ. In particular, the evaluated area was selected within areas that contain mitochondria, identified by MitoTracker Green staining. The SOX levels are presented as the percentage relative to the basal untreated HaCaT cells (basal control), which were set as 100%. All the experiments were conducted at least in triplicate.

### 2.12. Statistical Analysis

Data are expressed as mean ± SEM. Normality was assessed using the Shapiro-Wilk and Bartlett tests. As all variables followed a normal distribution, statistical comparisons were performed with one-way ANOVA followed by a Bonferroni post hoc test. Significance thresholds were set at *p* < 0.05 (*), *p* < 0.01 (**), and *p* < 0.001 (***). Analyses were conducted using GraphPad Prism 8.0 (GraphPad Software Inc., San Diego, CA, USA).

## 3. Results

### 3.1. Different Antioxidant Activity of WT and Cultivated Extracts by DPPH Assay

Aqueous extracts of wild-type (WT) and cultivated *P. nebrodensis* basidiomes were prepared as reported in Materials and Methods. Wild-type strains were designated as 1a, 2a, 3a, 1b, 2b, and 3b, while cultivated strains were designated as 3A, 2B(II), CAN1 °F, and CAN2 °F. Antioxidant activity was evaluated using the DPPH assay.

**Figure 1:**
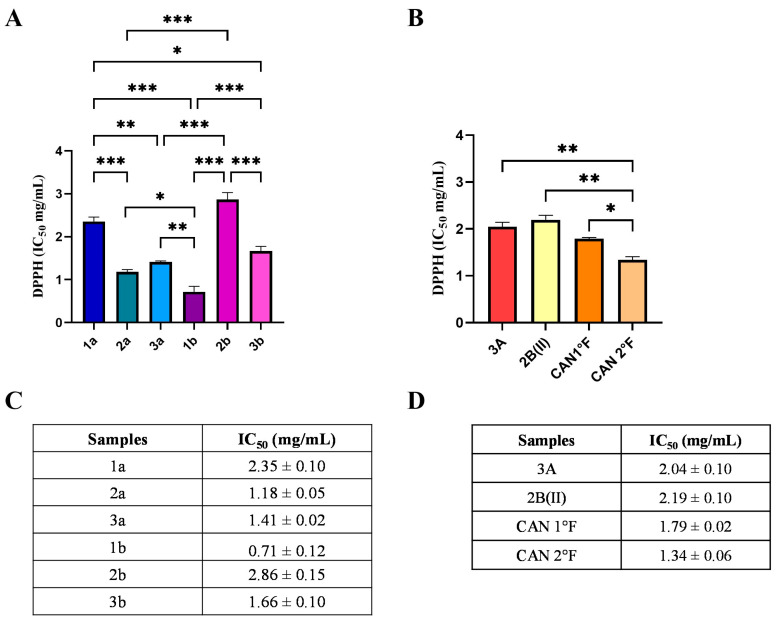
Chemical antioxidant activity of WT and cultivated samples assessed by the DPPH assay. IC_50_ values (mg/mL) of extracts from WT (panels **A**,**C**) and from cultivated (panels **B**,**D**) samples. Results are expressed as mean ± standard error of the mean (SEM). Statistically significant differences were determined using one-way ANOVA followed by Bonferroni’s post hoc test: *p* < 0.05 (*), *p* < 0.01 (**), *p* < 0.001 (***). *n* = 3 for each sample.

The results showed that all WT and cultivated extracts exhibited antioxidant activity (Figure 1), but to a different extent. IC_50_ values ranged from 0.71 to 2.86 mg/mL. Specifically, among WT, 1b sample showed the lowest statistically significant IC_50_ value (0.71 ± 0.12 mg/mL), thereby representing the most powerful extract in terms of radical scavenging activity (Figure 1A,C; statistical significance: 1b vs. 1a *p* < 0.001; 2a: *p* < 0.01; vs. 2b: *p* < 0.0001; vs. 3b: *p* < 0.001; vs. 3a: *p* < 0.001; Figure 1A). Extract 2a exhibited an IC_50_ value (1.18 ± 0.05 mg/mL) lower than those of samples 1a and 2b (vs. 1a: *p* < 0.001; vs. 2b: *p* < 0.0001), but, as previously mentioned, higher than that of 1b. IC_50_ values for samples 3a (1.41 ± 0.02 mg/mL) and 3b (1.66 ± 0.10 mg/mL) did not differ significantly from each other, although both were less effective than extracts 1b and 2a, and significantly more efficient than extracts 1a and 2b. Extracts from samples 1a and 2b were the least effective in terms of scavenging activity, as evidenced by their significantly higher IC_50_ values (1a: 2.35 ± 0.10 mg/mL; 2b: 2.86 ± 0.15 mg/mL) compared to all other samples. Taken together, these findings allow classification of the radical scavenging activity of WT extracts as follows: 1b > 2a > 3a > 3b > 1a > 2b.

Regarding cultivated samples, CAN2 °F showed the lowest IC_50_ value (1.34 ± 0.06 mg/mL), representing the most effective cultivated extract in terms of radical scavenging activity (Figure 1B,D). The IC_50_ value of CAN2 °F was significantly different from all other samples (vs. 3A: *p* < 0.01; vs. 2B(II): *p* < 0.01; vs. CAN1 °F: *p* < 0.05; Figure 1B). By contrast, extracts 3A (IC_50_: 2.04 ± 0.09 mg/mL), 2B(II) (IC_50_: 2.19 ± 0.09 mg/mL), and CAN1 °F (IC_50_: 1.79 ± 0.06 mg/mL) displayed comparable scavenging activities, with no statistically significant differences among them. The DPPH assay allowed the selection of the six most effective antioxidant extracts: 1b, 2a, 3a, 3b, CAN1 °F, and CAN2 °F, discarding the four extracts that exhibited the IC_50_ values higher than 2 mg/mL.

### 3.2. Evaluation of P. nebrodensis Extracts on Cell Viability Through MTT Assay

**Figure 2:**
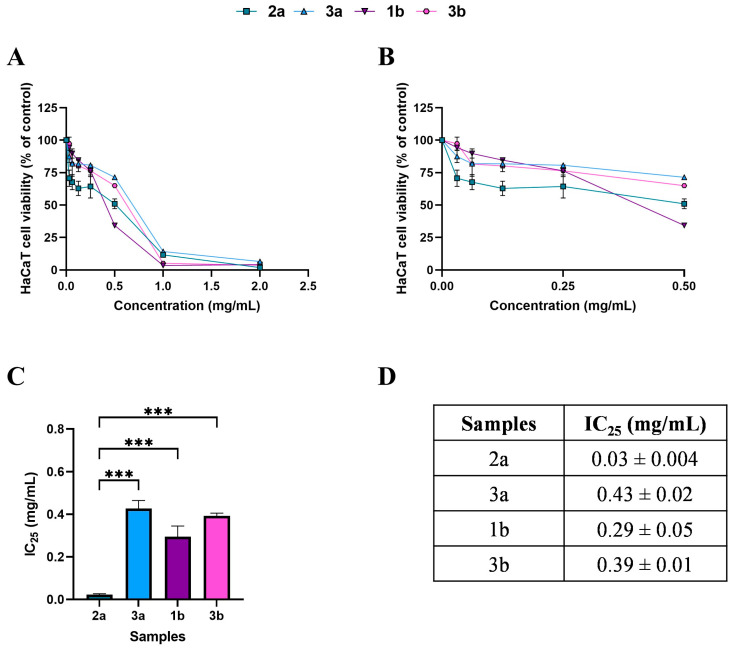
Effects of 24 h treatment with WT samples 2a (square), 3a (triangle), 1b (inverted triangle), and 3b (hexagon), across a concentration range of 0–2 mg/mL, on HaCaT cell viability (panel (**A**)). Enlargement of panel A in the 0–0.5 mg/mL concentration range (panel (**B**)). Histogram (panel (**C**)) and table (panel (**D**)) reporting the mean extract concentrations that maintain 75% cell viability (IC_25_). Results are expressed as mean ± standard error of the mean (SEM). Statistically significant differences were determined using one-way ANOVA followed by Bonferroni’s post-hoc test: *p* < 0.001 (***). *n* = 3 for each sample.

**Figure 3:**
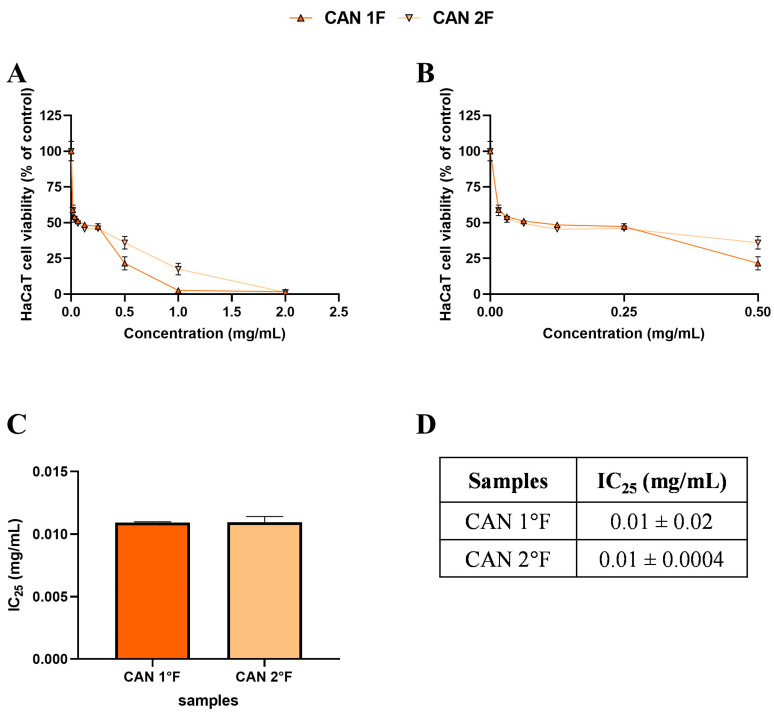
Effects of 24 h treatment with cultivated samples CAN1 °F (triangle) and CAN2 °F (inverted triangle) across a concentration range of 0–2 mg/mL on HaCaT cell viability (panel (**A**)). Enlargement of panel **A** within the 0–0.5 mg/mL concentration range (panel (**B**)). Histogram (panel (**C**)) and table (panel (**D**)) report the mean extract concentrations that maintain 75% cell viability (IC_25_). Results are expressed as mean ± standard error of the mean (SEM). Statistically significant differences were determined using one-way ANOVA followed by Bonferroni’s post-hoc test ((**C**) and (**D**), respectively). *n* = 3 for each sample.

After the DPPH extract selection, we tested the antioxidant activity in vitro using HaCaT cells, an immortalized human keratinocyte cell line. By MTT assay, we tested different concentrations of each fungal extract to identify the 75% cell viability (or IC_25_) subtoxic dose to avoid any cytotoxic effect on cells. Figure 2 and Figure 3 illustrate the effects of treatment with WT and cultivated extracts (at concentrations ranging from 0 to 2 mg/mL) for 24 h on cell viability.

Based on the MTT, subtoxic concentrations, corresponding to 75% cell viability, were selected for the subsequent antioxidant assay (Figure 2C,D and Figure 3C,D). It should be noted that, among WT samples, extract 2a exhibited approximately ten-fold higher biological activity on the HaCaT cells, reaching the IC_25_ value at a very low concentration of 0.03 mg/mL, which was statistically different compared to 3a (*p* < 0.0001), 1b (*p* < 0.0001), and 3b (*p* < 0.0001) extracts. In contrast, the IC_25_ values of 3a, 1b, and 3b extracts were comparable, with no statistically significant differences observed. Interestingly, the comparable and low IC_25_ values of the cultivated samples, CAN1 °F and CAN2 °F, suggested a very high biological activity of the two samples on the HaCaT cells.

### 3.3. Prevention of Induced Oxidative Stress in HaCaT Cells

After the selection of the DPPH assay, and after establishing the subtoxic effect, the preventive ROS-related antioxidant effect of extracts 1b, 2a, 3a, 3b, CAN1 °F, and CAN2 °F was further investigated using the cytological DCFH-DA assay in HaCaT cells (for details, see Section 2.8 in Materials and Methods). Each extract was tested at the IC_25_ dose determined through the MTT assay.

**Figure 4:**
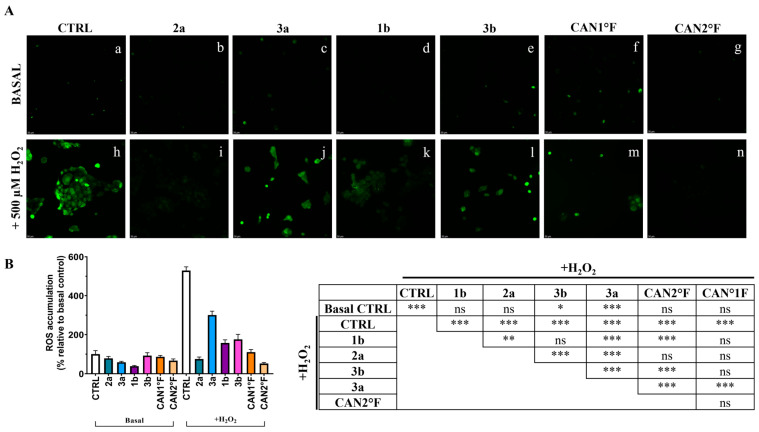
Evaluation of the antioxidant activity of WT samples (2a, 3a, 1b, and 3b) and cultivated samples (CAN1 °F and CAN2 °F) in HaCaT cells through DCFH-DA assay. (panel (**A**)) Representative DCFH-DA images under basal conditions (top, a–g) and after oxidative stress induction with H_2_O_2_ (500 µM, bottom, h–n). (panel (**B**)) ROS accumulation % relative to basal control (left) and statistical significance (right). Results are expressed as mean ± standard error of the mean (SEM). Statistically significant differences (one-way ANOVA followed by Bonferroni’s post-test): *ns* non-significant, *p* < 0.05 (*), *p* < 0.01 (**), *p* < 0.001 (***). *n* = 4 for each sample.

As shown in Figure 4, the treatment with the six extracts for 24 h did not significantly (*p* > 0.05) affect the low basal ROS levels observed in the CTRL condition in HaCaT cells. Exposure to 500 μM hydrogen peroxide (H_2_O_2_) induced a strong oxidative stress response, with ROS levels increasing more than fivefold relative to basal CTRL (*p* < 0.0001). Pretreatment for 24 h with all extracts effectively counteracted this increase. All extracts showed a significant reduction in % ROS accumulation compared to the H_2_O_2_ CTRL (*p* < 0.0001; Figure 4B). Furthermore, cells pretreated with 2a, 1b, CAN1 °F, and CAN2 °F displayed ROS levels comparable to the basal non-induced CTRL condition, indicating that the four extracts were able to prevent the oxidative stress induction. Conversely, pretreatment with 3a and 3b only partially reduced induced ROS accumulation, as ROS levels remained significantly higher than basal CTRL (vs. 3a: *p* < 0.0001; vs. 3b: *p* = 0.022). Among the different extracts in the H_2_O_2_ condition, 2a and CAN2 °F extracts exhibit the highest ROS inhibition compared to the other extracts (*p* < 0.0001), followed by CAN1 °F, 1b, and 3b, which induced significantly greater ROS inhibition compared to 3a (*p* < 0.0001).

### 3.4. Comparison of the Antioxidant Activity in Chemical and Cell Culture Assay

Subsequently, all results obtained from the DPPH and DCFH-DA assays (after H_2_O_2_ induction) were compared by normalizing each result to the highest value measured in the respective assay (Table 4). The sum of the percentage effects was calculated in Table 4. The cultivated sample CAN2 °F exhibited the highest antioxidant capacity, followed by the WT samples 1b and 2a. Furthermore, sample 1b showed stronger activity in the chemical (DPPH) assay than in the cytological (DCFH-DA) assay, whereas sample 2a displayed comparable effects in both assays. Samples 3a and 3b (WT), and CAN1 °F (cultivated) exhibited lower antioxidant activity compared to the other samples. These results allowed us to select CAN2 °F and 1b as the two samples with the highest overall antioxidant activity.

### 3.5. Correlation Between Glucan Content and Antioxidant Activity

**Figure 5:**
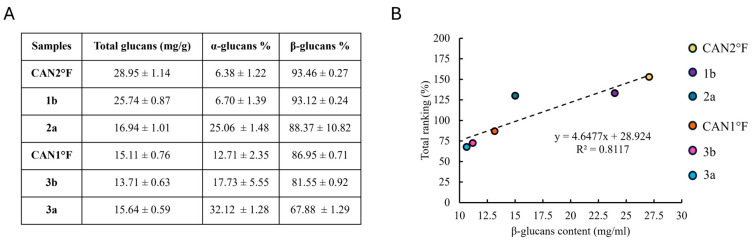
Glucan content and its correlation with antioxidant activity. (panel (**A**)) Content of total glucans (mg/g) and the percentage of α-glucans and β-glucans in the six selected samples. Results are expressed as mean ± standard error of the mean (SEM) (*n* = 4 for each sample). (panel (**B**)) Relationship between total ranking (%) and β-glucans content (mg/mL) in the six selected samples.

Considering that water extraction is enriched in β-glucans and that glucans have been previously reported to possess antioxidant properties, we quantified the glucan content in the extracts for which the total ranking was calculated. Accordingly, we quantified the total, α-, and β-glucan contents in CAN2 °F, 1b, 2a, CAN1 °F, 3b, and 3a extracts. Figure 5A shows the total glucan content (mg/g) and the percentage of α-glucans and β-glucans. Next, we evaluate whether a correlation exists between antioxidant activity and β-glucans content, and, notably, we found a linear relationship (R^2^ = 0.81) between the antioxidant activity and the β-glucans content (Figure 5B).

### 3.6. Prevention of Induced Mitochondrial Superoxide Production in HaCaT Cells

**Figure 6:**
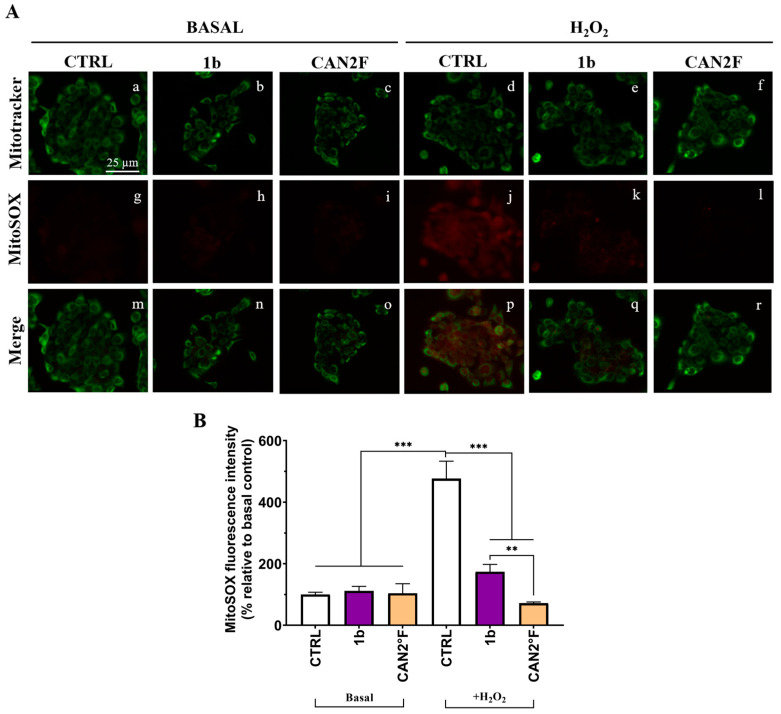
Effects of extracts 1b (WT) and CAN2 °F (cultivated) pretreatment on mitochondrial SOX in HaCaT cells. (panel (**A**)) Representative MitoTracker (green, a–f) and MitoSOX (red, g–l) images under basal conditions and after H_2_O_2_ exposure (500 μM, 1 h); merged images (m–r). Scale bar: 25 μm. (panel (**B**)) Histogram showing MitoSOX fluorescence intensity (% relative to basal control). Results are expressed as mean ± standard error of the mean (SEM). Statistically significant differences (one-way ANOVA followed by Bonferroni’s post-test): *p* < 0.01 (**), *p* < 0.001 (***). *n* = 3 for each sample.

We investigated the mechanism of the antioxidant effect in HaCaT cells by measuring mitochondrial superoxide (SOX) production using the MitoSOX™ Red assay. The study was focused on the most powerful antioxidant extracts, 1b and CAN2 °F, at the same IC_25_ dose determined by the MTT assay. As reported in Figure 6, the mitochondrial SOX level was negligible in the CTRL condition, and pretreatment for 24 h with both 1b and CAN2 °F extracts did not change the basal mitochondrial SOX level. The oxidative stress condition induced by 500 μM H_2_O_2_ significantly increased mitochondrial SOX production by nearly fivefold (*p* < 0.0001). Interestingly, pretreatment with both 1b and CAN2 °F for 24 h significantly reduced mitochondrial SOX levels under H_2_O_2_-induced oxidative stress, bringing SOX levels comparable to CTRL (*p* < 0.0001, Figure 6). Furthermore, it should be highlighted that CAN2 °F significantly reduced mitochondrial SOX level more effectively than 1b (*p* = 0.003).

### 3.7. Direct Scavenging Antioxidant Activity in HaCaT Cells

Up to this moment, we demonstrated that pretreatment with the different extracts prevents the H_2_O_2_-induced intracellular ROS and mitochondrial superoxide (SOX) production, suggesting that a mechanism of overproduction of antioxidant defense might be involved. We then investigated whether the two extracts could exert antioxidant effects during oxidative stress induction, which would imply direct scavenging antioxidant activity as a free radical scavenger.

**Figure 7:**
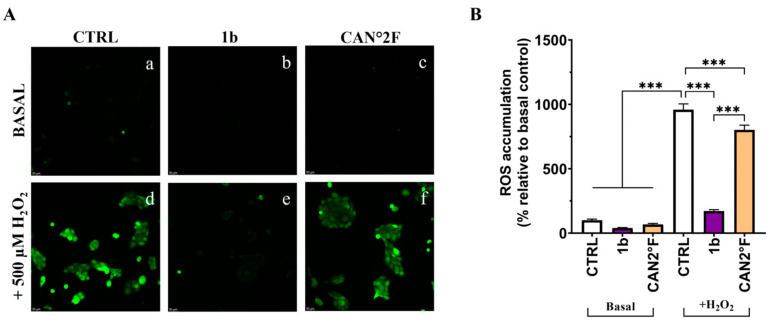
Direct scavenging antioxidant activity of WT (1b) and cultivated (CAN2 °F) samples in HaCaT cells evaluated by DCFH-DA assay. (panel (**A**), a–f) Representative DCFH-DA images under basal conditions (top) and after H_2_O_2_ exposure (500 µM, bottom). (panel (**B**)) ROS accumulation % relative to basal control. Results are expressed as mean ± standard error of the mean (SEM). Statistically significant differences (one-way ANOVA followed by Bonferroni’s post-test): *p* < 0.001 (***). *n* = 3 for each sample.

**Figure 8:**
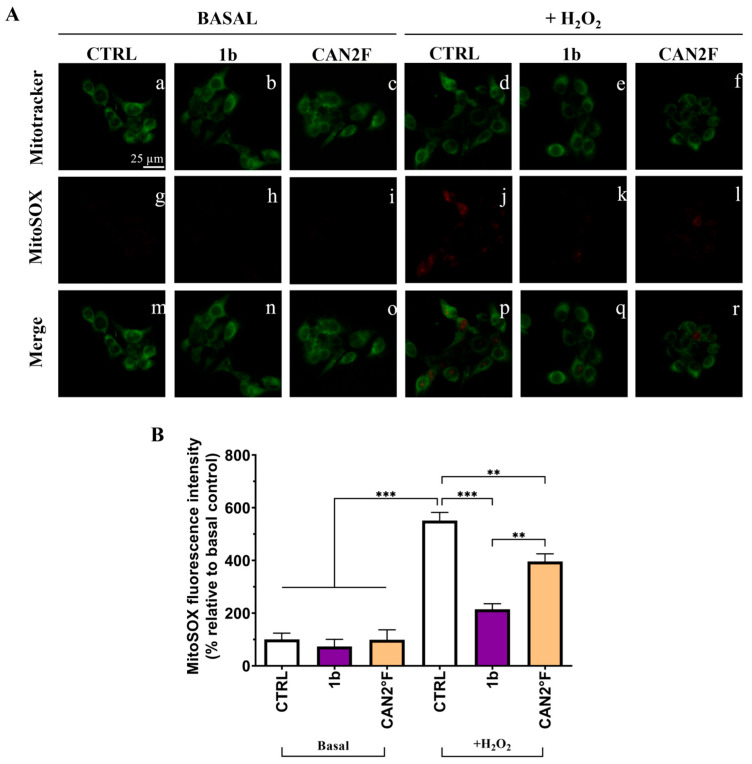
Direct scavenging antioxidant activity of WT (1b) and cultivated (CAN2 °F) samples in HaCaT cells evaluated by MitoSOX Red assay. (panel (**A**)) Representative MitoTracker (green, a–f) and MitoSOX (red, g–l) images under basal conditions and after H_2_O_2_ exposure (500 μM); merged images (m–r). (panel (**B**)) Histogram showing MitoSOX fluorescence intensity (% relative to basal control). Results are expressed as mean ± standard error of the mean (SEM). Statistically significant differences (one-way ANOVA followed by Bonferroni’s post-test): *p* < 0.01 (**), *p* < 0.001 (***). *n* = 4 for each sample.

As reported in Figure 7 and Figure 8, in basal condition, 30 min of treatment with 1b and CAN2 °F did not change the intracellular ROS and mitochondrial SOX levels (*p* > 0.05), evaluated through DCFH-DA and MitoSOX Red assay, respectively. Confirming the previous results, exposure to 500 μM H_2_O_2_ induced a strong oxidative stress response, increasing ROS and SOX levels by more than five-fold (*p* < 0.0001). Notably, co-treatment with 1b and H_2_O_2_ significantly decreased both intracellular ROS (*p* < 0.0001) and SOX (*p* < 0.0001) levels, bringing them back to levels comparable to those of untreated basal CTRL. Similarly, co-treatment with CAN2 °F significantly reduced intracellular ROS and mitochondrial SOX levels compared to H_2_O_2_ alone (*p* < 0.0001), but the reduction was significantly lower compared to that observed with 1b (*p* < 0.0001 for ROS and *p* < 0.01 for SOX). Thus, in this direct scavenging antioxidant activity evaluation, the 1b extract was more efficient in reducing ROS and SOX accumulation than the CAN2 °F extract (Figure 7 and Figure 8).

## 4. Discussion

Oxidative stress is closely linked to skin health, influencing not only aging processes but also the onset of various dermatological disorders [2,3,4]. Therefore, the search for effective natural strategies to protect keratinocytes is of growing interest, with implications extending beyond cosmetics to biomedicine. In this context, medicinal mushrooms represent a valuable source of antioxidant compounds, although the potential of *Pleurotus nebrodensis* remains unexplored.

In our study, we tested different aqueous extracts of both wild-type and cultivated *P. nebrodensis* basidiomes. The first assay, based on the spectrophotometric DPPH method, allowed the measurement of the direct scavenging antioxidant capacity of the different extracts and enabled the selection of the most promising samples. Specifically, the most promising samples were identified as 1b, 2a, 3a, and 3b for WT and CAN1 °F and CAN2 °F for the cultivated samples, discarding the four extracts that exhibited IC_50_ values higher than 2 mg/mL.

Subsequently, the antioxidant effect of the selected extracts was tested on HaCaT human keratinocytes at IC_25_ values, as determined by the MTT assay, both in basal and under H_2_O_2_-induced oxidative stress. Using the DCFH-DA and MitoSOX assays, we were able to evaluate (i) the preventive and indirect effects in reducing intracellular ROS and mitochondrial SOX accumulation, and (ii) the direct scavenging effect.

To study the preventive effect, HaCaT cells were pretreated with extracts for 24 h before oxidative stress induction by H_2_O_2._ The strongest antioxidant extract was CAN2 °F, as evidenced by the lower level of ROS accumulation. Under the same experimental conditions, CAN2 °F demonstrated the highest mitochondrial anti-SOX activity, suggesting that the preventive effect may originate from the mitochondria, possibly through the reinforcement of endogenous antioxidant systems. It is plausible that the preventive anti-ROS and anti-SOX activity of CAN2 °F may involve the activation of the Nrf2/ARE signaling pathway, one of the principal cellular mechanisms responsible for counteracting oxidative stress. Accordingly, after activation, Nrf2 translocates into the nucleus and binds to ARE sequences, promoting the transcription of several antioxidant enzymes, including superoxide dismutase (SOD), catalase (CAT), and heme oxygenase-1 (HO-1) [21]. The upregulation of these enzymes strengthens the intracellular defense and enhances the ability of keratinocytes to neutralize ROS and SOX before damage occurs [22]. While the present study does not directly assess these pathways, the observed effects of the extracts are consistent with antioxidant mechanisms reported for other medicinal mushrooms that activate Nrf2-dependent cytoprotection and modulate mitochondrial redox balance [23].

Furthermore, the direct scavenging antioxidant activity, investigated by spectrophotometric DPPH assay, was confirmed in HaCaT cells by the co-treatment of the two extracts with the oxidative stress inducer H_2_O_2_ in the DCFH-DA and MitoSOX Red assays. Selected extracts (1b and CAN2 °F) significantly reduced the oxidative stress, but 1b was the most powerful one, restoring ROS and SOX levels to values comparable to those of untreated controls.

In summary, CAN2 °F primarily exerts its activity by upregulating the antioxidant defense state of the cell, counteracting ROS and SOX production, whereas 1b primarily functions as a direct scavenger of ROS and SOX. This speculation is inferred from the results of the DCFH-DA and MitoSOX cellular assays in pretreatment conditions, evaluating the preventive antioxidant effect and direct scavenging activities in extract-H_2_O_2_ co-treatment conditions, and the DPPH assay. However, as previously anticipated, the limitation of the current study needs future investigations that should investigate targeted enzymatic or molecular analysis (e.g., assessing the Nrf2 pathway or quantifying the expression of specific antioxidant enzymes) to confirm the proposed mechanisms.

The glucan analysis revealed that both WT and cultivated extracts contain comparable amounts of total, α-, and β-glucans, with β-glucans representing the predominant fraction. These values are consistent with previously reported glucan contents in other medicinal and edible mushrooms [24,25]. β-glucans contribute substantially to antioxidant activity through both direct free radical scavenging and the modulation of endogenous cellular defenses in skin [26,27]. In more detail, our results suggest that, although the two selected extracts contain comparable levels of β-glucans, the underlying cellular mechanisms are different: a primarily direct scavenging activity for 1B and/or an antioxidant system modulation for CAN2 °F. Crucially, these two distinct mechanisms do not appear to be related to the nature of the extracts (wild-type or cultivated), as suggested by the variable effects observed among the samples.

Furthermore, by extending the β-glucans content analysis to additional extracts, a positive correlation emerged between the amount of β-glucans and the total antioxidant activity, suggesting that β-glucans were at least partially responsible for the observed biological activity. While β-glucans are a primary class of active constituents by water extraction, we cannot exclude that other bioactive hydrophilic compounds (such as phenolic compounds, triterpenes, or vitamins) present contribute to the overall activity. This suggests that the final antioxidant action is the result of an interaction between multiple constituents, rather than a single compound class. Indeed, synergistic interactions among phenolic compounds have been widely documented to enhance antioxidant activity [28], and the role of phytochemicals in modulating antioxidant defenses has been described in mixed phytochemical systems, such as fungal extracts [29]. Accordingly, the chemical characterization performed in this study is restricted to glucan quantification, and we cannot rule out the possibility that additional hydrophilic bioactive constituents may also contribute to the observed activity. These results are in line with previous research on medicinal mushrooms, which have been shown to exert antioxidant effects in skin cells through scavenging of free radicals, upregulation of endogenous antioxidant enzymes, and protection of mitochondrial integrity [30,31]. In particular, the activity of water extracts highlights the importance of hydrophilic compounds in antioxidant activity, a role already well documented in the literature [32]. Similar results have been described for *Auricularia auricula-judae* (Bull.) Quél., *Ganoderma lucidum* (Curtis) P. Karst., and *Pleurotus flabellatus* Sacc., where mushroom extracts reduced ROS accumulation, improved fibroblasts or keratinocytes viability, and promoted skin recovery [33,34].

To our knowledge, this is the first study demonstrating the antioxidant activity of *P. nebrodensis* extracts in human keratinocytes, showing their ability to reduce oxidative and mitochondrial stress, and linking their functional effects to glucan content. These findings provide a foundation for future mechanistic investigations and highlight the potential of *P. nebrodensis* as a candidate for cosmeceutical applications.

## 5. Conclusions

This survey demonstrates that selected *P. nebrodensis* aqueous extracts exert a strong preventive antioxidant effect and/or a significant direct free radical scavenging activity in human keratinocytes. Specifically, the prevention of induced oxidative stress is mirrored by a reduction in mitochondrial superoxide, thereby protecting cells against oxidative challenges. These findings support their potential as natural antioxidants for skin protection, with possible applications in preventing oxidative stress-related damage, aging, inflammation, and inflammaging conditions. To our knowledge, this is the first evidence of antioxidant activity of *P. nebrodensis* in human skin cells, expanding the repertoire of mushroom-derived bioactives with relevance for dermatological and cosmeceutical research. β-glucans are believed to be the predominant metabolites obtained through hot-water extraction, and our results show a positive correlation between β-glucans content and antioxidant activity. Importantly, this study also highlights that 1b and CAN2 °F extracts act through distinct antioxidant mechanisms, one predominantly associated with direct scavenging capacity and the other with modulation of endogenous cellular defenses. This raises the hypothesis that combining the two extracts into a blend may yield a pleiotropic effect, providing complementary antioxidant functions and potentially enhancing overall cytoprotective efficacy. Moving forward, further studies are warranted to isolate and characterize the active constituents, as well as to clarify the molecular pathways involved in their cytoprotective effects, including potential modulation of endogenous antioxidant–cellular antioxidant pathways.

## Figures and Tables

**Table 1:** Correspondence between wild-type strains and the cultivated strains derived from them.

CODE Wild-Type Strain	CODE Cultivated Strain
1a–1b	CAN1 °F
2a–3a	3A
2b	2B(II)
3b	CAN2 °F

**Table 2:** Codes of the wild-type basidiomes, initial weight, weight after lyophilization, and extraction yield.

Samples	Dried Basidiomes Weight (g)	Weight After Lyophilization (mg)	Extraction Yield %
1a	1	395.12	39.51
2a	1	379.38	37.94
3a	1	337.41	33.74
1b	1	330.53	33.05
2b	1	340.78	34.08
3b	1	402.01	40.20

**Table 3:** Codes of the cultivated samples, initial weight, weight after lyophilization, and extraction yield.

Samples	Dried Basidiomes Weight (g)	Weight After Lyophilization (mg)	Extraction Yield %
3A	1	387.23	38.72
2B(II)	1	429.28	42.93
CAN1 °F	1	497.52	49.75
CAN2 °F	1	266.02	26.60

**Table 4:** Normalized score and comparison between the chemical DPPH assay and the cytological DCFH-DA assay for the six selected samples.

Samples	DPPH %	DCFH-DA %	Total Activity %
CAN2 °F	52.99	100.00	152.99
1b	100.00	33.22	133.22
2a	60.17	69.66	129.83
CAN1 °F	39.66	47.29	86.96
3b	42.77	29.71	72.49
3a	50.35	17.35	67.70

## Data Availability

The original contributions presented in this study are included in the article. Further inquiries can be directed to the corresponding authors.

## Abbreviations

The following abbreviations are used in this manuscript:

ROS	Reactive oxygen species
SOX	Superoxide
DPPH	2,2-diphenyl-1-picrylhydrazyl
MTT	3-(4,5-dimethylthiazol-2-yl)-2,5-diphenyltetrazolium bromide
PBS	Phosphate-buffered saline
DCFH-DA	Dichloro-dihydro-fluorescein diacetate
CR	Critically Endangered
Di.S.S.P.A.	Department of Soil, Plant and Food Sciences
POWO	Plants of the World Online
PDA	Potato dextrose agar
DMSO	Dimethyl sulfoxide
GOPOD	Glucose oxidase-peroxidase
IC50	Half maximal inhibitory concentration
IC25	Concentration necessary to inhibit 25% of cell viability
CTRL	Control
H2O2	Hydrogen peroxide
WT	Wild-type
